# Comparison of the Clinical Implications among Five Different Nutritional Indices in Patients with Lupus Nephritis

**DOI:** 10.3390/nu11071456

**Published:** 2019-06-27

**Authors:** Sung Soo Ahn, Juyoung Yoo, Seung Min Jung, Jason Jungsik Song, Yong-Beom Park, Sang-Won Lee

**Affiliations:** 1 Division of Rheumatology, Department of Internal Medicine, Yonsei University College of Medicine, Seoul 03722, Korea; 2 Institute for Immunology and Immunological Diseases, Yonsei University College of Medicine, Seoul 03722, Korea

**Keywords:** lupus nephritis, end-stage renal failure, nutrition, index, prognosis

## Abstract

Systemic lupus erythematosus (SLE) is characterized with aberrant responses in the immune systems and lupus nephritis (LN) is one of the most serious complications of SLE. This study evaluated the clinical significance of different nutritional indices in 207 renal biopsy-proven LN patients. The clinical and laboratory data were reviewed, and five different nutritional indices were calculated: (i) Controlling nutritional status (CONUT) score; (ii) prognostic nutritional index (PNI); (iii) nutritional risk index; (iv) neutrophil-to-lymphocyte ratio; and (v) body mass index. The factors associated with end-stage renal failure (ESRF) were assessed using a Cox-proportional hazard analysis. The patients with ESRF had significantly lower median PNI (31.1 vs. 34.7, *p* = 0.012) than those without ESRF at baseline. The CONUT score and PNI had the highest correlation between the SLE disease activity index-2000 (*r* = 0.467 and *p* = −0.356, all *p* < 0.001) and was significantly associated with SLE activity-related measures. In the Cox-proportional hazard analysis, PNI (odds ratio 0.925, 95% confidence interval 0.865–0.989, *p* = 0.022) was independently associated with ESRF along with creatinine and chronicity index, and the renal survival rate was significantly lower in patients with PNI ≤35.41 than in those with PNI >35.41 (*p* = 0.003). Among nutritional indices, the CONUT score and PNI better correlated with disease activity and PNI was associated with ESRF.

## 1. Introduction

Systemic lupus erythematosus (SLE) is a prototypical autoimmune disease, with a characteristic feature of aberrant responses in the innate and adaptive immune system [1]. These abnormalities in the immune system contribute to the development of autoimmunity in SLE, damaging various organs. Regarding the organs involved in SLE, lupus nephritis (LN) is one of the most serious complications which is associated with a high morbidity and mortality [2]. It is estimated that, during the course of SLE, nearly 60% of patients develop LN, among whom 10% eventually develop end-stage renal failure (ESRF) [3,4]. Therefore, it is important to identify patients highly susceptible to ESRF. The ultimate goal in treating LN is to prevent ESRF through appropriate immunosuppressive treatment. Accordingly, numerous efforts have been made to discover the factors associated with patient prognosis in LN.

In general, malnutrition is considered to be a condition associated with increased morbidity and mortality [5]. Meanwhile, compromised nutritional status was reported to be associated with chronic inflammation as well as the development of autoimmunity [6]. Therefore, investigations have been made to elucidate the prognostic implication of the nutritional status in various diseases. Interestingly, some studies have indicated that abnormalities in the nutritional status are associated with a poor prognosis in malignancies, as well as autoimmune diseases, such as rheumatoid arthritis (RA) and systemic sclerosis. This suggests that assessment of malnutrition could provide clinically valuable information regarding patient prognosis [7,8,9].

Traditionally, anthropometric measures, such as the height or body mass index (BMI) or laboratory tests, i.e., albumin, prealbumin, and total cholesterol level in the blood, have been used to assess the individual’s nutritional status [10,11]. Nonetheless, because there is no common consensus regarding the most useful tool to assess the nutritional status, inconsistencies abound regarding the prognostic implication of nutritional status in various diseases. Recently, mini-nutritional indices including the controlling nutritional status (CONUT) score, prognostic nutritional index (PNI), nutritional risk index (NRI), and neutrophil-to-lymphocyte ratio (NLR) have also been increasingly used as surrogate markers to reflect the patients’ nutritional status [12,13,14]. Given that SLE is a representative systemic inflammatory autoimmune disease, it could be speculated that nutritional indices at diagnosis could be associated with patient prognosis in SLE. However, the predictive potential of nutritional indices for the prognosis of LN is still unknown. Hence, the aim of this study was to (i) investigate the association between 5 different nutritional indices with disease activity, and (ii) evaluate whether these nutritional indices are associated with the development of ESRF in patients with biopsy-proven LN during the follow-up.

## 2. Materials and Methods

### 2.1. Patient Inclusion

The medical records of 260 patients who had undergone renal biopsy between August 2005 and August 2018 and were diagnosed with LN were reviewed. From the 260 patients included, this study excluded 53 for the following reasons: (i) Patients who did not fulfil the 1997 revised American College of Rheumatology (ACR) classification criteria for SLE [15,16]; (ii) patients with histopathologic findings incompatible to the criteria proposed by the 2003 International Society of Nephrology/Renal Pathology Society (ISN/RPS) [17,18]; (iii) in LN patients who had undergone repeat renal biopsy, only the result of the first biopsy was used. Finally, a total of 207 patients were included. This study was approved by the Institutional Review Board of Severance Hospital and conducted in accordance with the principles set forth in the Declaration of Helsinki, and the requirement to obtain informed consent was waived because of the retrospective study design (4-2018-1083).

### 2.2. Clinical and Laboratory Data Collection

The authors obtained the clinical and laboratory data of the date when the pathologic diagnosis of LN was made. The demographic data collected included age and sex. For SLE activity-related measures, the following were evaluated: The SLE disease activity index-2000 (SLEDAI-2K); white blood cell (WBC) and platelet counts; the levels of complement (C)3, C4, and anti-dsDNA; and the urinary protein/creatinine ratio (P/Cr), which is a laboratory component of SLEDAI-2K [19]. The clinical features of patients consisted of skin rashes, photosensitivity, oral ulcers, arthritis, serositis, neurologic, hematologic, and immunologic disorders, according to the 1997 ACR classification criteria [15]. Other laboratory data included lymphocyte count, erythrocyte sedimentation rate (ESR), and levels of C-reactive protein (CRP), creatinine, total cholesterol, serum albumin, aspartate aminotransferase (AST), and alanine aminotransferase (ALT). The glomerular filtration rate (GFR) was calculated by the chronic kidney disease epidemiology collaboration study equation [20]. Concerning renal biopsy data, the class of LN as well as the activity and chronicity indices were assessed based on the 2003 ISN/RPS classification criteria. The medication usage after the diagnosis of LN was assessed using the Korean drug utilization system.

### 2.3. Nutritional Indices Selection and Calculation

Five different measures of nutritional indices were included in the study: CONUT score, PNI, NRI, NLR, and BMI. The calculations of CONUT score, PNI, NRI, NLR, and BMI were performed according to the following formulae: (i) CONUT score is the sum of serum albumin score, total lymphocyte count score, and total cholesterol score based on a predefined cut-off (Supplementary Table S1) [21]; (ii) PNI is 10 × serum albumin value (g/dL) + 0.005 × total lymphocyte count in the peripheral blood (/mm^3^) [22]; (iii) NRI is (1.519 × serum albumin (g/dL)) + (41.7 × weight (kg)/ideal body weight (kg)) [13]; (iv) NLR is the total neutrophil count in the peripheral blood/total lymphocyte count in the peripheral blood (/mm^3^) [23]; (v) BMI is weight (kg)/height (m^2^). Being overweight was defined as a BMI of over 23.0, in accordance with the Asia-Pacific guidelines of obesity classification [24].

### 2.4. Statistical Analysis

All statistical analyses were performed using MedCalc statistical software version 18.11 (MedCalc Software, Ostend, Belgium). The continuous variables were presented as median and interquartile ranges, and categorical variables were presented as frequencies and percentages. For the comparison of continuous and categorical variables, the Mann-Whitney U test and Kruskal-Wallis test, and the chi-square or Fisher’s exact test were used as appropriate. The correlation between nutritional indices and variables were assessed using the Pearson’s correlation analysis. Low and high PNI was defined using receiver operating characteristics (ROC) curve and patients with PNI ≤35.41 were classified as having low PNI, while patients with PNI >35.41 were classified as having high PNI. Kaplan-Meier analysis was used to compare the renal survival rate of patients with low and high PNI. Univariable and multivariable Cox-proportional hazard analysis was used to evaluate factors associated with ESRF. In all statistical analysis, a two-tailed *p* < 0.05 was considered to be significant.

## 3. Results

### 3.1. Clinical Characteristics of Patients

The baseline characteristics of patients included in the study are described in Table 1. The median age was 36.0 years and 186 (89.9%) of the patients were female. The median follow-up duration was 57.1 months. When the patients were divided into those with and without ESRF during the follow-up, 20 (9.7%) were classified as having ESRF, while 187 (90.3%) were classified as without. Regarding SLE activity-related measures, the patients with ESRF had significantly lower platelet counts and anti-dsDNA titres. There was no significant difference in the clinical features between the groups. In addition, the patients with ESRF presented with lower lymphocyte counts and GFR but higher creatinine levels at diagnosis. For renal biopsy data, mixed class V LN was observed more frequently in patients without ESRF, whereas the chronicity index was significantly higher in patients with ESRF. In addition, when the authors compared the nutritional indices between the groups, differences were found only in PNI, and patients with ESRF had significantly lower PNI than those without (median PNI 31.1 vs. 34.7, *p* = 0.012). No significant differences were noted regarding the medications that were administered in both groups during the follow up (Supplementary Table S2).

### 3.2. Correlation between Variables and Nutritional Indices

When the association between variables and nutritional indices were evaluated, SLE disease activity-related measures, such as SLEDAI-2K, WBC and platelet count, C3, C4, and anti-dsDNA levels, and urinary P/Cr ratio, were significantly correlated with the CONUT score and PNI. SLEDAI-2K had the highest correlation with CONUT score, followed by PNI (*r* = 0.467, and *r* = −0.356, all *p* < 0.001). Although SLEDAI-2K and C3 were correlated with NRI, the association was weak. Moreover, significant correlations between the WBC count and the NLR were found, and urinary P/Cr ratio was associated with BMI among SLE activity-related measures (Table 2).

Concerning other variables, the lymphocyte count was significantly correlated with the CONUT score, PNI, and NLR. The CRP was significantly correlated with the CONUT score, PNI, NLR, and BMI. Creatinine levels only correlated with PNI, while GFR only correlated with BMI. The total cholesterol level was correlated with the PNI, NLR, and BMI and the serum albumin level was found to be correlated with the CONUT score, PNI, and NRI. Lastly, AST level was correlated only with the CONUT score and the activity index seen on renal biopsy was correlated only with the CONUT score and PNI (Table 2). On evaluating the associations with the nutritional indices, the CONUT score was correlated with the PNI, NRI, and NLR (*r* = −0.780, *r* = −0.199, and *r* = 0.304, respectively). In addition, the PNI was significantly correlated with NRI and NLR (*r* = 0.165, *p* = 0.018 and *r* = −0.272, *p* < 0.001) and the NRI with BMI (*r* = 0.861, *p* < 0.001) (Supplementary Table S3).

### 3.3. Comparison of Different Nutritional Indices According to Lupus Nephritis Subclasses

**Figure 1:**
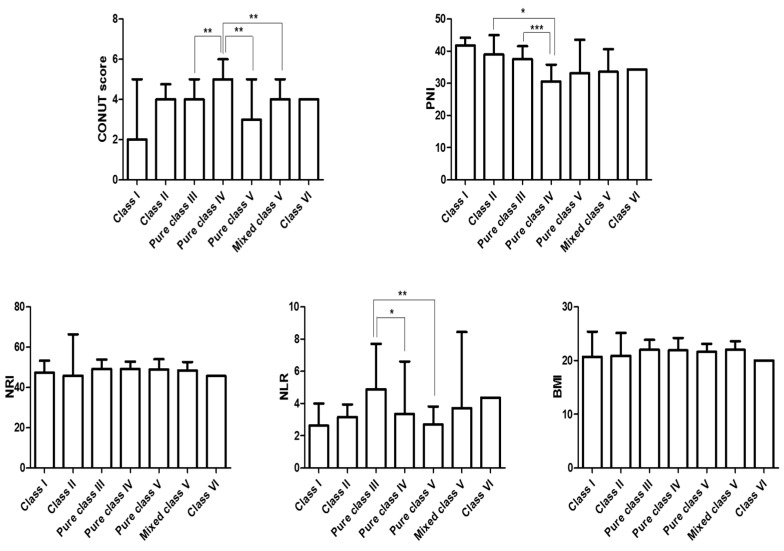
The comparison of different nutritional indices according to the lupus nephritis subclasses at diagnosis. CONUT, Controlling nutritional status; PNI, Prognostic nutritional index; NRI, Nutritional risk index; NLR, Neutrophil to lymphocyte ratio; BMI, Body mass index. *p < 0.05, **p < 0.01, ***p < 0.001 between the groups.

**Figure 2:**
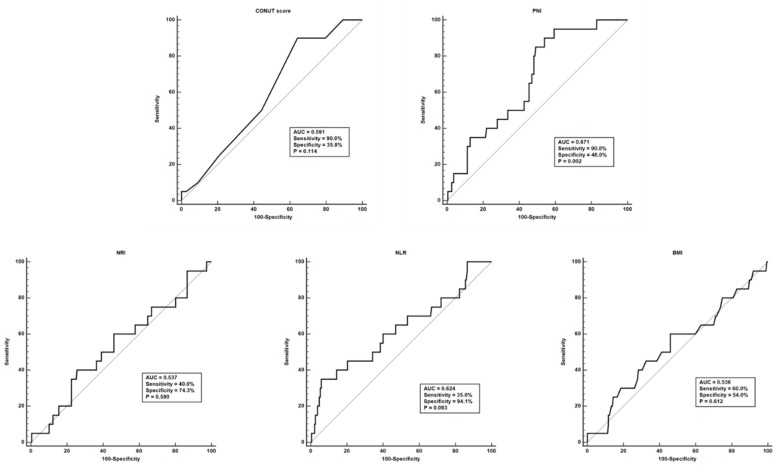
Receiver operator characteristic curve of nutritional indices at lupus nephritis diagnosis in predicting end-stage renal failure. CONUT, Controlling nutritional status; AUC, Area under the curve; PNI, Prognostic nutritional index; NRI, Nutritional risk index; NLR, Neutrophil to lymphocyte ratio; BMI, Body mass index.

Next, this study compared whether there was any difference in the nutritional indices included based on LN subclasses. The patients with pure class IV LN had a significantly higher CONUT score than patients with pure class III and V LN, as well as those with mixed class V LN. In addition, the PNI was significantly lower in patients with pure class IV LN than in those with class II and pure class III LN (Figure 1). Furthermore, patients with pure class III and class IV LN had a higher NLR than those with pure class V nephritis. However, no significant difference was found regarding the NRI and BMI according to LN classes (Figure 1). On the other hand, among the 5 nutritional indices included, only PNI was capable of predicting ESRF (area under the ROC curve = 0.671, sensitivity 90.0%, specificity 46.0%, 95% confidence interval (CI) 0.602–0.734, *p* = 0.002), and the optimal cut-off of PNI in predicting ESRF was determined to be PNI ≤35.41 (Figure 2).

### 3.4. Factors Associated with End-stage Renal Failure in Patients with Lupus Nephritis

**Figure 3:**
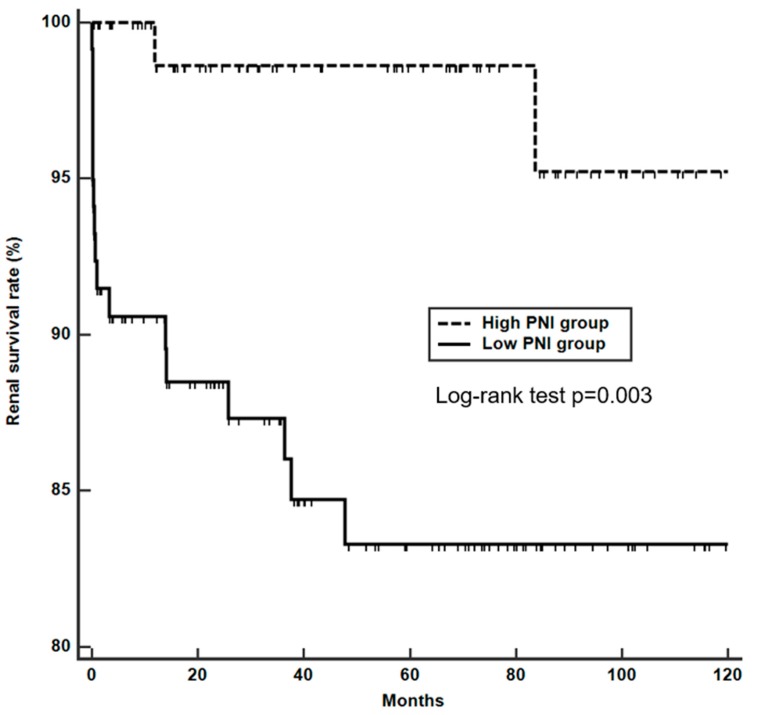
The Kaplan-Meier curve analysis of renal survival rate according to PNI at lupus nephritis diagnosis. PNI, Prognostic nutritional index.

This study performed a Cox-proportional hazard analysis to elucidate the factors associated with ESRF. In univariable analysis, the platelet and lymphocyte count, creatinine, GFR, chronicity index, PNI, and NLR were significantly associated with ESRF. However, in multivariable analysis, only creatinine (odds ratio (OR) 1.623, 95% CI 1.322–1.993, *p* < 0.001), chronicity index (OR 1.458, 95% CI 1.203–1.767, *p* < 0.001), and PNI (OR 0.925, 95% CI 0.865–0.989, *p* = 0.022) were independent factors for ESRF (Table 3). The Kaplan-Meier analysis also revealed that patients with a low PNI had a significantly higher probability of having ESRF than those with a high PNI (log rank test *p* = 0.003) (Figure 3).

## 4. Discussion

To the best of the authors knowledge, the present study is the first to evaluate the clinical significance of 5 different nutritional indices in patients with LN. Among the included indices, the CONUT score and PNI were better correlated with SLE disease activity-related measures to assess disease activity in LN. However, only the PNI was significantly lower in patients with ESRF at LN diagnosis. On the Cox-proportional hazard analysis, the PNI was an independent predictor of ESRF along with creatinine and chronicity index. Our observations imply that among the nutritional indices, the CONUT score and PNI could be useful to assess disease activity. However, only PNI was seen to have prognostic implications in identifying patients with LN at a high risk of developing ESRF.

The findings of our study indicate that the CONUT score and PNI could be useful markers to assess both global and localized disease activity in patients with LN. Notably, the correlation analysis between nutritional indices and SLE activity-related measures revealed that the CONUT score and PNI were better correlated with the SLEDAI-2K, which is the most widely used index to assess global SLE disease activity, along with WBC and platelet counts, C3, C4, and anti-dsDNA levels, urinary P/Cr ratio (which is a laboratory component of the SLEDAI-2K), and the activity index as determined through renal biopsy. Moreover, in patients with pure class IV LN, which is the most devastating form of LN, the CONUT score and PNI were found to be significantly different compared to those of other subclasses.

The CONUT score and PNI were first developed to estimate undernutrition and the risk of postoperative complications in patients with gastrointestinal cancers, respectively. However, accumulating evidences have identified that they are also associated with the prognosis of patients with other malignancies and disorders related to malnourishment [25,26,27,28]. The association of the CONUT score and PNI with SLE disease activity at LN diagnosis that was found in this study could be explained by the fact that both indices are calculated using the lymphocyte count in the peripheral blood and serum albumin level. Albumin is a negative acute phase protein that could decrease in proportion to systemic inflammatory response and the hallmark of LN is proteinuria [29]. Therefore, it is possible that both systemic and localized inflammation could contribute to the development of hypoalbuminemia. Moreover, lymphopenia has been included as a hematologic criterion according to the 1997 ACR and 2012 SLE International Collaborating Clinics SLE criteria [15,30] and has been reported to be associated with SLE disease activity and prognosis [31]. Similar findings were found in previous studies in which PNI was significantly correlated with SLE disease activity independent of other factors, supporting the results of our study [32,33].

Owing to the poor prognosis of LN, numerous attempts have been made to evaluate the prognostic factors associated with LN. Regarding histopathological factors, the presence of proliferative LN and higher activity and chronicity indices at diagnosis have been proposed to be associated with a poor prognosis [34]. Contreras et al., and Singh et al., also demonstrated that higher creatinine and chronicity index was a predictor of poor prognosis in LN [35,36]. Notably, our data revealed that the PNI was an independent factor associated with ESRF in multivariate Cox-proportional hazard analysis, along with creatinine and chronicity index in renal biopsy during the follow-up period. Additionally, the renal survival rate in patients with low PNI was significantly lower than those with high PNI, implying that PNI could provide additional information regarding the prediction of ESRF in LN. However, the authors found that the presence of proliferative LN and activity index were not significantly associated with ESRF, which could be associated with the disease-modulating effect of the immunosuppressive agents administered.

Among nutritional indices included, our study indicated that only PNI was capable of assessing renal outcome in patients with LN. Surprisingly, because the CONUT score had the highest correlation with SLEDAI-2K and laboratory variables included in SLEDAI-2K at baseline, the association between PNI and ESRF, instead of the CONUT score, was rather unexpected. However, the discordant results between disease activity and the development of ESRF could be attributed to the fact that baseline disease activity is not a predictive factor for patient prognosis in SLE [37]. In contrast, it has been reported that persistent proteinuria, which could lead to hypoalbuminemia as a consequence of albumin loss, as well as the serum albumin level itself, were indicative of renal outcomes in LN [38,39,40]. Compared to the CONUT score in which a relatively equal weighting is given between serum albumin, lymphocyte count, and total cholesterol level, the PNI is mainly dependent on the serum albumin level. Considering the importance of the serum albumin level in LN, it could be reasoned that the PNI could serve as a more appropriate measure to assess renal outcomes than the CONUT score.

In the treatment of proliferative LN, current guidelines recommend treating patients with induction therapy using either cyclophosphamide or mycophenolate mofetil, followed by sustained maintenance therapy with mycophenolate mofetil or azathioprine [4]. When this study compared the treatment between patients with ESRF and without, there were no significant differences in the proportion of patients treated with glucocorticoids, cyclophosphamide, mycophenolate mofetil, and azathioprine, suggesting that the selection of induction and maintenance treatment might not have substantially affected the patients’ clinical outcomes.

BMI is the most widely used measure to assess obesity in the general population. A previous study has indicated that the prevalence of obesity in patients with SLE was reported to range from 28–50% [41]. Similar findings were noted in our study population, as the proportion of overweight patients (36.2%) was not significantly different. Interestingly, several reports have suggested that obesity is associated with disease development and poor outcomes in patients with autoimmune diseases. Previous studies have shown that obesity was associated with an increased risk of RA, SLE, and psoriatic arthritis (PsA) [42,43,44]. In addition, it has been suggested that increased weight was related to a lower possibility of sustained remission in patients with early RA and was related to higher disease activity and diminished clinical response in patients with PsA [45,46]. Obesity has also been suggested to be associated with an increased risk of cardiovascular disease in patients with SLE [47]. Although first recognized solely as a measure of body composition, obesity is now regarded as a mild chronic inflammatory state that could affect the immune system via adipose-derived inflammation. In addition, previous studies have suggested that high BMI is associated with increased proteinuria and albuminuria [48]. Accordingly, a significant correlation was observed between BMI, CRP, and urinary P/Cr ratio in the present study. However, in this study, BMI itself did not correlate with SLEDAI-2K and the proportion of patients developing ESRF during the follow-up did not differ between the groups, implying that BMI may not be an appropriate measure to assess disease activity and the risk of ESRF in LN.

The strength of this study is that, to the best of the authors’ knowledge, this is the first study that evaluated the clinical significance of different nutritional indices in patients with LN. However, several limitations were also present in our study. First, the assessment of clinical and laboratory data was performed by reviewing patients’ medical records. Second, the number of patients with ESRF included was relatively small. Third, serial changes in the nutritional indices were not assessable and the adjustment for treatment was not possible owing to the retrospective study design. Fourth, it remains uncertain if albumin replacement therapy could be beneficial in improving the prognosis in patients with LN. Additional studies are required to validate our findings and reveal the association between nutritional status and patient prognosis in LN.

## 5. Conclusions

In conclusion, the authors have demonstrated that among the nutritional indices examined in this study, the CONUT score and PNI were better correlated with disease activity and the PNI was associated with ESRF in patients with LN. Both the CONUT score and PNI could be useful measures to assess disease activity; in particular, PNI may provide useful information in stratifying patients at high risk of ESRF in LN.

## Figures and Tables

**Table 1:** The baseline characteristics of patients at lupus nephritis diagnosis.

Variables	Total (*n* = 207)	Patients with ESRF (*n* = 20)	Patients without ESRF (*n* = 187)	*p*-value
**Demographic data**				
Age, years	36.0 (27.0–46.0)	37.5 (32.5–51.0)	36.0 (26.0–46.0)	0.210
Female sex, *n* (%)	186 (89.9)	19 (95.0)	167 (89.3)	0.700
Follow-up duration, months	57.1 (17.5–90.8)	33.3 (9.9–82.9)	59.2 (19.7–93.6)	0.214
**SLE activity-related measures**				
SLEDAI-2K	9.0 (7.0–12.0)	9.0 (5.0–11.0)	9.0 (7.0–13.0)	0.079
WBC count (/mm^3^)	4560.0 (3337.5–6805.0)	4390.0 (2795.0–7755.0)	4560.0 (3372.5–6650.0)	0.767
Platelet count (×1000/mm^3^)	205.0 (138.3–251.8)	183.5 (68.5–222.0)	207.0 (145.8–254.0)	0.036
Complement 3, mg/dL	45.6 (29.5–69.1)	42.1 (27.5–59.5)	45.9 (29.6–70.8)	0.374
Complement 4, mg/dL	6.2 (3.2–13.3)	6.8 (3.8–12.7)	6.2 (3.1–13.3)	0.995
Anti-dsDNA (IU/mL)	174.8 (10.3–379.0)	25.0 (0.0–245.2)	196.9 (20.3–379.0)	0.042
Urinary P/Cr ratio	2.9 (1.5–6.1)	4.2 (1.5–6.9)	2.8 (1.5–5.9)	0.422
**Clinical features, *n* (%)**				
Skin rash	55 (26.6)	4 (20.0)	51 (27.3)	0.601
Photosensitivity	14 (6.8)	0 (0.0)	14 (7.5)	0.370
Oral ulcer	22 (10.6)	1 (5.0)	21 (11.2)	0.702
Arthritis	11 (5.3)	0 (0.0)	11 (5.9)	0.605
Serositis	48 (23.2)	6 (30.0)	42 (22.5)	0.449
Neurologic disorder	2 (1.0)	0 (0.0)	2 (1.1)	0.999
Hematologic disorder	179 (86.5)	18 (90.0)	161 (86.1)	0.999
Immunologic disorder	183 (88.4)	17 (85.0)	166 (88.8)	0.710
**Laboratory data**				
Lymphocyte count (/mm^3^)	900.0 (602.5–1260.0)	655.0 (330.0–1070.0)	940.0 (610.0–1270.0)	0.012
ESR (mm/h)	48.0 (26.0–75.0)	46.0 (28.0–66.5)	48.0 (26.0–75.0)	0.867
CRP (mg/L)	2.3 (1.0–7.3)	4.7 (1.7–15.8)	2.3 (1.0–6.3)	0.061
Cr (mg/dL)	0.8 (0.6–1.1)	1.6 (0.9–3.6)	0.8 (0.6–1.1)	<0.001
GFR (CKD-EPI), mL/min/1.73 m^2^	94.0 (64.3–115.0)	38.5 (17.0–73.5)	100.0 (69.0–117.0)	<0.001
Total cholesterol (mg/dL)	196.0 (159.3–243.8)	220.0 (185.0–234.5)	195.0 (156.0–245.8)	0.557
Serum albumin (g/dL)	2.9 (2.3–3.4)	2.5 (2.1–3.1)	2.9 (2.3–3.4)	0.067
AST (IU/L)	21.0 (17.0–35.5)	26.0 (16.5–44.5)	21.0 (17.0–34.0)	0.366
ALT (IU/L)	16.0 (10.0–25.0)	15.0 (9.5–30.0)	16.0 (10.0–25.0)	0.684
**Renal biopsy data**				
**Lupus nephritis class, *n* (%)**				
Class I	3 (1.4)	0 (0.0)	3 (1.6)	0.999
Class II	8 (3.9)	0 (0.0)	8 (4.3)	0.999
Pure class III	49 (23.7)	5 (25.0)	44 (23.5)	0.883
Pure class IV	92 (44.4)	13 (65.0)	79 (42.2)	0.052
Pure class V	23 (11.1)	1 (5.0)	22 (11.8)	0.706
Mixed class V	31 (105.0)	0 (0.0)	31 (16.6)	0.049
Class V + II	1 (0.5)	0 (0.0)	1 (0.5)	
Class V + III	21 (10.1)	0 (0.0)	21 (11.2)	
Class V + IV	9 (4.3)	0 (0.0)	9 (4.8)	
Class VI	1 (0.5)	1 (5.0)	0 (0.0)	0.097
**Activity/Chronicity index**				
Activity index	7.0 (2.0–11.0)	8.5 (4.5–12.0)	7.0 (2.0–11.0)	0.126
Chronicity index	1.0 (1.0–2.0)	2.0 (1.5–5.0)	1.0 (1.0–2.0)	<0.001
**Nutritional indices**				
CONUT score	4.0 (3.0–5.0)	4.5 (4.0–5.5)	4.0 (3.0–5.0)	0.173
PNI	33.9 (26.7–39.3)	31.1 (23.7–34.1)	34.7 (27.0–39.9)	0.012
NRI	48.8 (44.4–53.2)	49.8 (44.5–53.8)	48.7 (44.4–53.0)	0.584
NLR	3.7 (2.1–6.7)	4.7 (2.4–12.4)	3.6 (2.1–6.3)	0.070
BMI	21.9 (20.0–24.0)	22.4 (20.1–25.1)	21.8 (19.9–23.9)	0.595

The values are expressed as the median (interquartile range) or *n* (%). ESRF, End-stage renal failure; SLE, Systemic lupus erythematosus; SLEDAI-2K, Systemic lupus erythematosus disease activity index-2000; WBC, White blood cell; P/Cr, Protein/creatinine; ESR, Erythrocyte sedimentation rate; CRP, C-reactive protein; Cr, Creatinine; GFR, Glomerular filtration rate; CKD-EPI, Chronic kidney disease epidemiology collaboration; AST, Aspartate aminotransferase; ALT, Alanine aminotransferase; CONUT, Controlling nutritional status; PNI, Prognostic nutritional index; NRI, Nutritional risk index; NLR, Neutrophil to lymphocyte ratio; BMI, Body mass index.

**Table 2:** Correlation analysis between variables and nutritional indices at lupus nephritis diagnosis

Variables	CONUT Score	PNI	NRI	NLR	BMI
**SLE activity-related measures**					
SLEDAI-2K	0.467 (<0.001)	−0.356 (<0.001)	−0.183 (0.008)	−0.091 (0.192)	−0.088 (0.206)
WBC count (/mm^3^)	−0.264 (<0.001)	0.205 (0.003)	0.053 (0.449)	0.548 (<0.001)	0.089 (0.200)
Platelet count (×1000/mm^3^)	−0.329 (<0.001)	0.260 (<0.001)	0.063 (0.371)	0.072 (0.302)	−0.002 (0.978)
Complement 3, mg/dL	−0.502 (<0.001)	0.377 (<0.001)	0.138 (0.047)	0.058 (0.403)	0.086 (0.217)
Complement 4, mg/dL	−0.335 (<0.001)	0.223 (0.001)	0.083 (0.236)	0.052 (0.454)	0.024 (0.737)
Anti-dsDNA (IU/mL)	0.278 (<0.001)	−0.144 (0.038)	−0.046 (0.508)	−0.089 (0.201)	0.007 (0.919)
Urinary P/Cr ratio	0.221 (0.001)	−0.515 (<0.001)	0.031 (0.655)	0.064 (0.361)	0.151 (0.030)
**Laboratory data**					
Lymphocyte count (/mm^3^)	−0.662 (<0.001)	0.549 (<0.001)	0.102 (0.145)	−0.400 (<0.001)	0.057 (0.413)
ESR (mm/hr)	0.101 (0.148)	−0.105 (0.134)	−0.095 (0.173)	−0.109 (0.119)	−0.090 (0.198)
CRP (mg/L)	0.246 (<0.001)	−0.141 (0.043)	0.059 (0.401)	0.166 (0.017)	0.137 (0.049)
Cr (mg/dL)	0.109 (0.118)	−0.153 (0.028)	0.021 (0.762)	0.097 (0.166)	0.100 (0.153)
GFR (CKD-EPI), mL/min/1.73 m^2^	−0.042 (0.550)	0.118 (0.090)	−0.130 (0.062)	−0.084 (0.228)	−0.216 (0.002)
Total cholesterol (mg/dL)	−0.133 (0.056)	−0.274 (<0.001)	0.091 (0.192)	0.144 (0.038)	0.165 (0.018)
Serum albumin (g/dL)	−0.611 (<0.001)	0.922 (<0.001)	0.147 (0.034)	−0.135 (0.053)	−0.055 (0.433)
AST (IU/L)	0.191 (0.006)	−0.097 (0.164)	−0.091 (0.190)	0.010 (0.887)	−0.111 (0.110)
ALT (IU/L)	0.037 (0.597)	0.031 (0.658)	0.002 (0.981)	0.032 (0.646)	−0.034 (0.631)
**Renal biopsy data**					
Activity index	0.188 (0.007)	−0.215 (0.002)	−0.007 (0.921)	0.072 (0.302)	0.053 (0.448)

The data are shown in correlation coefficient followed by *p*-value in parentheses. CONUT, Controlling nutritional status; PNI, Prognostic nutritional index; NRI, Nutritional risk index; NLR, Neutrophil to lymphocyte ratio; BMI, Body mass index; SLE, Systemic lupus erythematosus; SLEDAI-2K, Systemic lupus erythematosus disease activity index-2000; WBC, White blood cell; P/Cr, Protein/creatinine; ESR, Erythrocyte sedimentation rate; CRP, C-reactive protein; Cr, Creatinine; GFR, Glomerular filtration rate; CKD-EPI, Chronic kidney disease epidemiology collaboration; AST, Aspartate aminotransferase; ALT, Alanine aminotransferase.

**Table 3:** The Cox-proportional hazard analysis of variables associated with end-stage renal failure during the follow-up.

	Univariable Analysis			Multivariable Analysis		
Variables	Odds Ratio	95% Confidence Interval	*p*-Value	Odds Ratio	95% Confidence Interval	*p*-Value
SLEDAI-2K	0.904	0.803–1.016	0.091			
WBC count (/mm^3^)	1.000	0.999–1.000	0.744			
Platelet count (×1000/mm^3^)	0.993	0.987–0.999	0.015			
Complement 3, mg/dL	0.992	0.974–1.010	0.362			
Complement 4, mg/dL	0.995	0.938–1.054	0.853			
Anti-dsDNA (IU/mL)	0.998	0.995–1.000	0.063			
Urinary P/Cr ratio	1.078	0.964–1.206	0.185			
Lymphocyte count (/mm^3^)	0.999	0.998–0.999	0.023			
ESR (mm/h)	1.000	0.986–1.014	0.999			
CRP (mg/L)	1.012	0.999–1.025	0.070			
Cr (mg/dL)	1.827	1.522, 2.193	<0.001	1.623	1.322–1.993	<0.001
GFR (CKD-EPI), mL/min/1.73 m^2^	0.959	0.944, 0.975	<0.001			
Total cholesterol (mg/dL)	0.999	0.994–1.006	0.977			
Serum albumin (g/dL)	0.569	0.311–1.041	0.067			
AST (IU/L)	1.002	0.996–1.009	0.491			
ALT (IU/L)	0.999	0.986–1.014	0.979			
Pure proliferative lupus nephritis^¶^	4.300	0.998–18.534	0.050			
Activity index	1.076	0.989–1.171	0.089			
Chronicity index	1.493	1.254–1.778	<0.001	1.458	1.203–1.767	<0.001
CONUT score	1.230	0.957–1.582	0.106			
PNI	0.932	0.880–0.987	0.017	0.925	0.865–0.989	0.022
NRI	1.007	0.959–1.057	0.789			
NLR	1.070	1.020–1.122	0.006			
BMI	1.029	0.908–1.167	0.651			

^¶^Proliferative lupus nephritis was defined as either class III or IV lupus nephritis according to the International Society of Nephrology/ Renal Pathology Society criteria. SLEDAI-2K, Systemic lupus erythematosus disease activity index-2000; WBC, White blood cell; P/Cr, Protein/creatinine; ESR, Erythrocyte sedimentation rate; CRP, C-reactive protein; Cr, Creatinine; GFR, Glomerular filtration rate; CKD-EPI, Chronic kidney disease epidemiology collaboration; AST, Aspartate aminotransferase; ALT, Alanine aminotransferase; CONUT, Controlling nutritional status; PNI, Prognostic nutritional index; NRI, Nutritional risk index; NLR, Neutrophil to lymphocyte ratio; BMI, Body mass index.

## Supplementary Materials

The following are available online at https://www.mdpi.com/2072-6643/11/7/1456/s1, Table S1. Calculation of the CONUT score, Table S2. Comparison of medications administered in patients with and without end stage renal failure during the follow-up, Table S3. Correlation analysis between nutritional indices at lupus nephritis diagnosis.
