# Supplementary material for: Comparison of the Clinical Implications among Five Different Nutritional Indices in Patients with Lupus Nephritis
Source: Nutrients. 2019 Jun 27;11(7):1456. doi: 10.3390/nu11071456 (PMC6682980; doi:10.3390/nu11071456)
Supplement: Supplementary file 1 [file nutrients-11-01456-s001.pdf]

Supplementary Table S1. Calculation of the CONUT score

Laboratory parameters	None	Light	Moderate	Severe
Serum albumin (g/dL)	≥ 3.50	3.00-3.49	2.50-2.99	<2.50
Score	0	2	4	6
Total lymphocyte count (/mm3)	≥ 1600	1200-1599	800-1199	<800
Score	0	1	2	3
Total cholesterol (mg/dL)	≥ 180	140-179	100-139	<100
Score	0	1	2	3
CONUT score= Serum albumin score + total lymphocyte count score + total cholesterol score				

CONUT, Controlling nutritional status.

Supplementary Table S2. Comparison of medications administered in patients with and without end stage renal failure during the follow-up

Variables	Total (n=207)	Patients with ESRF (n=20)	Patients without ESRF (n=187)	p-value
**Medications, n (%)**				
Glucocorticoids	205 (99.0)	20 (100.0)	185 (98.9)	0.999
Hydroxychloroquine	112 (54.1)	7 (35.0)	105 (56.1)	0.072
Cyclophosphamide	67 (32.4)	8 (40.0)	59 (31.6)	0.444
Mycophenolate mofetil	161 (77.8)	13 (65.0)	148 (79.1)	0.149
Azathioprine	28 (13.5)	3 (15.0)	25 (13.4)	0.739
Tacrolimus	50 (24.2)	8 (25.0)	42 (22.5)	0.082
Cyclosporine	10 (4.8)	1 (5.0)	9 (4.8)	0.999

Values are expressed as n (%).

ESRF, End-stage renal failure.

Supplementary Table S3. Correlation analysis between nutritional indices at lupus nephritis diagnosis

Variables	CONUT score	PNI	NRI	NLR	BMI
CONUT score	n/a				
PNI	-0.780 (<0.001)	n/a	n/a	n/a	n/a
NRI	-0.199 (0.004)	0.165 (0.018)	n/a	n/a	n/a
NLR	0.304 (<0.001)	-0.272 (<0.001)	-0.105 (0.132)	n/a	n/a
BMI	-0.069 (0.326)	-0.024 (0.731)	0.861 (<0.001)	-0.054 (0.442)	n/a

Data are shown in correlation coefficient followed by p-value in parentheses.

CONUT, Controlling nutritional status; PNI, Prognostic nutritional index; NRI, Nutritional risk index; NLR, Neutrophil to lymphocyte ratio; BMI, Body mass index.
